# Healthier Lipid Profiles of Japanese Adults, Especially in Women with Elevated High-Density Lipoprotein Cholesterol (HDL-C), Are Associated with Low HDL-C Peroxide Content

**DOI:** 10.3390/antiox13121434

**Published:** 2024-11-22

**Authors:** Loni Berkowitz-Fiebich, Shelby M. Flaherty, Shinobu Kitayama, Mayumi Karasawa, Norito Kawakami, Attilio Rigotti, Christopher L. Coe

**Affiliations:** 1 Departamento de Nutricion Diabetes y Metabolismo, Potificia Universidad Catolica de Chile, Santiago 8320165, Chile; lberkowi@uc.cl (L.B.-F.); arigotti@med.puc.cl (A.R.); 2 Department of Surgery, Duke University, Durham, NC 27708, USA; shelby.flaherty@duke.edu; 3 Department of Psychology, University of Michigan, Ann Arbor, MI 48109, USA; kitayama@umich.edu; 4 Department of Comparative Psychology, Tokyo Woman’s Christian University, Tokyo 167-004, Japan; mayumik@lab.twcu.ac.jp; 5 Department of Mental Health, University of Tokyo, Tokyo 113-8654, Japan; norito@m.u-tokyo.ac.jp; 6 Department of Psychology, University of Wisconsin-Madison, 1202 West Johnson Street, Madison, WI 53706, USA

**Keywords:** Japan, high-density lipoprotein cholesterol, HDL peroxide content, aging, sex, obesity, lipid peroxidation

## Abstract

Japanese adults typically have healthier lipid profiles than American and European adults and a lower prevalence and later onset of atherosclerotic cardiovascular disease (ASCVD). Many Japanese also have uniquely elevated levels of high-density lipoprotein cholesterol (HDL-C). The following analysis examined the relationship between HDL-C level and HDL-C peroxide content, a bioindicator of unhealthy lipid metabolism in Japanese adults. Blood samples were collected from 463 participants, 31–84 years of age, who lived in Tokyo. A second blood sample was collected 5 years later from 241 of the participants, allowing us to evaluate the temporal stability of the inverse correlation between HDL-C level and HDL-C peroxide content. Glucoregulation and inflammatory activity were assessed because both can be associated with dyslipidemia and HDL-C dysfunction. Obesity and central adiposity were also considered. Overall, women had healthier HDL-C profiles than men. Elevated HDL-C (>90 mg/dL) was common (16.6%) and found more often in women. Higher HDL-C peroxide content was associated with older age and central adiposity and incremented further when HA1c and CRP were higher. When assessed 5 years later, lower HDL-C peroxide content continued to be evident in adults with higher HDL-C. While similar associations have been described for other populations, most Japanese adults typically had healthier levels of HDL-C with lower HDL-C peroxide content than previously reported for American adults.

## 1. Introduction

Ever since the seven countries study was initiated in the 1950s, it has been known that there is a lower prevalence of atherosclerotic cardiovascular disease (ASCVD) in Japan than in many other countries, and that most Japanese adults typically have healthier lipid profiles than found in other populations [1,2,3]. Some health benefits can be attributed to the Japanese diet and the relatively low occurrence of obesity [4,5,6]. In addition, there are national programs that encourage engagement in physical activity, including walking, and an effective universal health care delivery system. However, there is also evidence for a genetic basis for some population-level differences in lipid physiology. Many studies have documented that lipid profiles are already distinct in young Japanese children [7,8]. Similarly, while cholesterol levels change when Japanese adults reside in other countries and eat the local cuisine, their cholesterol values and disease risks continue to differ from the indigenous populations [9,10,11,12,13]. Historically, elevated levels of low-density lipoprotein cholesterol (LDL-C) were considered the primary risk factor for the pathogenesis of ASCVD [14,15]. However, there is now an increased awareness of the importance of the atheroprotective benefits of HDL-C and the deleterious consequences of having low levels of HDL-C [16]. Many Japanese adults have higher levels of HDL-C than typically seen in other populations [17,18,19]. The following analysis was designed to further characterize the lipid profiles of middle-aged and older adult residents of Tokyo with a focus on HDL-C. This assessment provided an opportunity to examine whether the benefits of higher HDL-C would increase in a linear and progressive manner or if there would be a biphasic association with health and HDL-C peroxide content when HDL-C levels were very elevated [20,21,22]. Studies of European and American adults have indicated that elevated HDL-C levels may be associated with an increased hazard risk for all-cause mortality, suggestive of dysfunctional HDL-C, although the specific link to ASCVD is usually more evident when HDL-C is low [23,24].

The anti-atherogenic benefits of HDL-C are due primarily to its involvement in the efflux of cholesterol from peripheral tissues and the reverse transport of this excess cholesterol to the liver for excretion by the gut, which limits deposition on the vasculature and reduces lipid accumulation in activated macrophages. HDL-C also has additional anti-oxidative and anti-inflammatory functions, moderating the oxidation of LDL-C [25]. HDL-C can become dysfunctional with the onset of Type 2 diabetes and fatty liver disease, and its beneficial actions can be undermined by unhealthy lifestyles, such as excessive alcohol consumption, and by increased inflammatory activity [26,27,28]. The health-promoting actions of HDL-C are associated with its particle size and composition, which can be impacted by oxidative stress, leading to lipid peroxidation and a build-up of reactive oxygen species, hydrogen peroxides, and superoxides [29,30,31]. One novel feature of the biomarker panel employed in our analyses was the inclusion of an assay to index lipid peroxidation by determining the peroxide content of HDL-C. This cell-free, fluorometric method was developed originally to evaluate the side effects of anti-viral treatments on cholesterol structures and levels in HIV-infected patients [32]. It was also shown to sensitively reflect improvements in lipid metabolism in obese patients after bariatric surgery and weight loss [33]. The protocol was then adapted for high throughput processing of sera to examine the peroxide content of HDL-C in a survey of middle-aged and older Americans [34]. That study showed that HDL-C peroxide content was associated with the adverse effects of obesity, diabetes, and aging on lipid metabolism. 

The primary aim of the following analysis was to evaluate the lipid and health profiles of adult Japanese residents of Tokyo and to investigate the influence of age and sex. The effects of adiposity, poor glycemic control and increased inflammatory activity were also examined as possible mediators of the variation in lipid profiles. In addition, a further goal was to explore the potentially distinctive aspects of lipid physiology in Japanese adults who had particularly high HDL-C levels. There have been many prior reports on Japanese adults with HDL-C elevated above 80–90 mg/dL [35,36], which were linked to the actions of genes associated with cholesteryl ester transfer protein (CETP). CETP transfers cholesterol from HDL-C to apolipoprotein-B, containing lipoproteins (i.e., VLDL and LDL) [36]. Patients with CETP deficiency were first identified in Japan in 1985 when examining individuals who had extremely high HDL-C [37]. Several studies have indicated that some CETP polymorphisms convey a risk for poor health, but the findings specifically on hypertension and ASCVD are inconsistent. Our analyses compared adults with HDL-C levels above 90 mg/dL to participants with HDL-C in the more typical range, specifically to assess the possible association with HDL-C peroxide content.

## 2. Methods

### 2.1. Participants

The 463 participants (247 female and 216 male) were a subset of 1684 middle-aged and older adults in the Midlife in Japan (MIDJA) study. They were recruited with random survey methods in 2008 and 2013 to represent the age and sex composition of the 23 residential wards of Tokyo (Table 1). Demographic variables and information about psychological measures and the use of prescription medications were obtained from all respondents by a questionnaire [38]. Individuals who consented to participate in the biomarker subproject provided blood samples at either a medical clinic or a laboratory at the University of Tokyo. The mean age of adults in this lipid profile analysis was 56.4 years (S.D. = 13.9 years, range: 31–84 years). Blood samples were also obtained 5 years later from 241 of the adults recruited in 2008, providing an opportunity to assess the stability of their lipid profiles. All procedures were approved by the Health Sciences Institutional Review Board at the University of Wisconsin as well as re-reviewed and approved by the Ethics Committee at the University of Tokyo (see IRB Statement and Informed Consent Statement for details).

### 2.2. Demographic Information and Prescription Medications

Age, sex, marital status, and educational attainment were determined for all participants. The average age and sex distribution in the biomarker subproject did not differ from that of the participants in the general survey. The statistical modeling of biological predictors of HDL-C peroxide content included a covariate for prescription medication use, based on the self-reporting of drugs and/or supplements that might have lipid-lowering actions (e.g., antihyperlipidemic agents, cholesterol absorption inhibitors, HMC-CoA reductase inhibitors, bile acid sequestrants, fibrates, niacin, and omega-3). Forty-eight of the 463 participants reported taking one or more of these medications and supplements.

### 2.3. Sample Collection

Blood was obtained from 247 women and 216 men for the biomarker assessments. Because many participants worked, it was not possible to obtain samples in the very early morning following an overnight fast. However, recent studies have shown that fasting has only a modest effect on total cholesterol and HDL-C test results, with the consumption of a recent meal primarily affecting triglyceride levels [39,40]. Over 95% of the samples were collected between 0900 and 1145, with most of the remainder by 1330. Only 8 samples were collected in the afternoon, all before 1530. During the visit, height and weight were measured and used to calculate body mass index (BMI, weight/height, kg/m^2^). Waist circumference was determined as an indicator of central adiposity. Systolic and diastolic blood pressure was also recorded during the visit.

### 2.4. Lipid Panel and HDL-C Peroxide Content

A traditional lipid panel, including total cholesterol, HDL-C, LDL-C, and triglyceride levels (mg/dL), was obtained at the clinical laboratory of the Showa University School of Medicine (Tokyo, Japan). In addition, frozen sera were stored in an ultracold freezer and shipped using an overnight courier on dry ice for analysis in the United States. One hundred lipid panels were ordered from a CLIA-certified clinical laboratory (Unity Health-Meriter Laboratory, Madison, WI, USA) to confirm that the lipid values determined in Tokyo were comparable to diagnostic testing in the U.S. (Figures S1 and S2).

The assays to determine HDL-C peroxide content were all conducted at one laboratory (Biomarker Core, Madison, WI, USA). The methods for generating this measure of lipid peroxidation have been published previously [32]. Briefly, a fixed volume protocol was adapted from the fluorescence assay used to assess HDL-C composition in patients, both lipid dysfunction in HIV-infected individuals on anti-viral medications, as well as the beneficial changes in obese adolescents after bariatric surgeries and weight loss [32,33]. Adaptations to optimize procedures for high throughput processing included the use of 96-well, Greiner polypropylene round bottom plates (Sigma-Aldrich, Milwaukee, WI, USA), and the inclusion of a positive control with purified HDL-C on each plate (Lee Biosolutions, Maryland Heights, MO, USA). The control HDL-C was maintained at a stock concentration of 2070 mg/dL and diluted to working concentrations of 300 mg/dL using 0.15 NaCl for each assay. Participant samples were assayed in triplicate on the same plate (CV < 0.5%). Sera were prepared by depleting apoB-containing particles via precipitation using dextran sulfate and magnesium chloride at concentrations of 2 μM and 50 mM, respectively (Millipore Sigma, Burlington, MA, USA). Fluorescence was measured with a Biotek Synergy H1 fluorimeter (Agilent, Santa Clara, CA, USA) at 530 nm and 590 nm wavelengths, and fluorescence at 2 h was used in the analysis. Quantification of HDL-C for the HDL-C positive control and ensuring HDL-C levels in the serum aliquots were concordant with the clinical laboratory value were performed with a WAKO Cholesterol E kit (Fujifilm Pure Chemical Corp., Tokyo, Japan), using a MRX^e^ DYNEX microplate reader (Magellan Biosciences, Chantilly, VA, USA). HDL peroxide content values were generated with the following calculations:

1. Fluorescence in sample minus fluorescence in blank = peroxidized HDL-C in participant sera, quantified in fluorescence units (FU).
2. FU/ HDL-C in sample = normalized peroxidized HDL-C per 1 mg/dL HDL-C (FU/mg HDL-C), the primary unit of quantification.
3. HDL-C peroxide content = normalized, peroxidized HDL-C per 1 mg/dL HDL-C in sera (FU per mg/dL HDL-C)/ standardized HDL-C peroxide content in a purified reference control. Steps 2 and 3 were performed with respect to the HDL-C level in the sera. The same pool of purified HDL-C was included in every assay as a common reference point for harmonizing values across assays given the large number of samples.

### 2.5. Additional Biomarkers

Glycosylated hemoglobin (HA1c) was determined from fresh, whole blood on the day of collection at the clinical laboratory of the Showa University School of Medicine (Tokyo, Japan). To ensure comparability with the diagnostic values generated by clinical laboratories in the U.S., aliquots from 10 blood samples were shipped overnight on cold refrigerant blocks and tested on arrival at the Unity Health-Meriter Laboratory (Madison, WI, USA). HA1c was determined by using a turbidimetric immunoinhibition assay. The test results from the two laboratories were highly correlated (*r* = 0.85, *p* < 0.01), but because the assay calibration scales were different, an algorithm was generated to calibrate the values to match HA1c testing in U.S. clinical studies (Figure S3). This adjustment did not change the relative ranking of HA1c values, nor alter the position on the continuum from low to high. A cutoff of 6.5% was used to identify individuals showing signs of insulin resistance and poor glycemic control [41]. 

Serum IL-6 levels were determined by a high-sensitivity enzyme-linked immunosorbent assay (Quantikine ELISA, R&D Systems, Minneapolis, MN, USA), with a lower sensitivity of detection at 0.16 pg/mL. All values were quantified in duplicate; values over 10 pg/mL were re-run in diluted sera to fall on the standard curve. The intra-assay coefficient of variance (CV) was 4.1%, and the inter-assay CV was 12.9%. High-sensitivity CRP was assessed with a particle-enhanced immunonepholometric assay at the Laboratory of Clinical Biochemistry Research (University of Vermont, Burlington, VT, USA). Briefly, polystyrene particles were coated with monoclonal antibodies to CRP, which resulted in an antigen agglutinate in the presence of CRP and increased light intensity, which was quantified with a BNII nephelometer (Seimans Healthcare Diagnostics, Deerfield, IL, USA). The assay range was 0.16–1100 mg/L; the intra-assay CV range was 2.3–4.4%, and the inter-assay CV ranged from 2.1 to 5.7%. 

### 2.6. Statistical Analysis

Descriptive statistics, including means and variance indices, were generated for all variables and examined for outliers. Predictors and endpoints evincing skewness were log-transformed to normalize the distribution. Data from all participants were retained to include the continuum from healthy to unhealthy and to ensure the findings were representative of the adult residents in Tokyo. Sex and age differences in predictor and endpoint variables were examined initially with two-way analyses of variance (ANOVA: female vs. male in 3 age categories, <50, 51–64, >65 years, respectively). To interrogate the primary predictors of HDL-C peroxide content, a series of six hierarchical regression models were run, sequentially adding in the following predictors: sex (male as reference), age, HA1c, CRP, waist circumference, and non-HDL-C. The possible influence of lipid-lowering medications and supplements that could affect HDL-C level and compositions was considered by including a binary covariate in the modeling (i.e., Yes/No for use of antihyperlipidemic agents, cholesterol absorption inhibitors, HMC-CoA reductase inhibitors, bile acid sequestrants, fibrates, niacin, omega-3, etc.). Differences in HDL-C peroxide content between adults with extremely high HDL-C and those with HDL-C in a more typical range were then compared by a 3-way ANOVA, including HDL-C level (<89 and >90 mg/dL), gender (female vs male) and age (3 levels: <50, 51–64, >65 years), as the between factors. For the participants who provided a second blood sample at follow-up 5 years later, variations in HDL-C and HDL-C peroxide content over time were evaluated with a repeated measures ANOVA. The lipid profiles and HDL-C peroxide content at the two time points were compared in the adults with lower and elevated levels of HDL-C. Pairwise comparisons were conducted with the Pearson’s *r* test. The alpha value for attaining statistical significance was set at <0.05.

## 3. Results

### 3.1. Descriptive Statistics

As shown in Table 2, the mean age of the participants at the initial recruitment was 56.4 years, with a range of 31–84 years. The adiposity measurements indicated that obesity with a BMI over 30, or even 25, was not common, but the assessment of waist circumference conveyed that central adiposity increased with age and was more evident in men than women. Mean systolic and diastolic blood pressures were also higher in males and evinced a similar trend to increase with age. Glycemic control, as reflected by glycosylated hemoglobin levels (HA1c), was considered as one of the biological predictors in the statistical modeling of HDL-C peroxide content. Based on HA1c values over 6.5% (>48 mmol/mol), and participant reports of physician-diagnosed diabetes, the prevalence of poor glycemic control was 7%. However, only seven female participants met these criteria, and they were older adults with a mean age of 66.9 years. Forty-eight of the 458 participants reported using prescription drugs and/or supplements that could have lipid-lowering effects. For that reason, medication use was included as a covariate in the regression models that examined predictors of HDL-C peroxide content. Participants taking medications were more likely to be older. Their mean age was 66.6 years as compared to 55.4 years for those not taking medications. It is also noteworthy that the HDL-C peroxide content was higher in the participants who reported taking lipid-lowering and/or hypertensive drugs (9.27 +/−0.63 vs. 7.83 +/−0.17, *p* < 0.007). Thus, these medications did not account for a reduction in the HDL-C peroxide content when HDL-C levels were high. Instead, the results suggested that higher HDL-C peroxide content was associated with poorer health. 

The two inflammatory proteins, CRP and IL-6, were typically low, but the circulating levels of both were significantly higher in men than women (Table 2). Because CRP and IL-6 levels were highly correlated (*r* = 0.57, *p* < 0.001), only CRP was included in the prediction modeling of HDL-C peroxide content. The lipid values of the MIDJA participants were generally in keeping with previous publications on middle-aged and older Japanese adults. Total cholesterol levels were high, often above 200 mg/dL, but HDL-C was a relatively large contributor. High levels of HDL-C moderated the proportion accounted for by non-HDL cholesterol. Although LDL-C values tended to be above norms reported for some other countries, they did not differ between women and men, nor did the level of LDL-C increase significantly with age. In contrast, the triglyceride levels were significantly higher in men than women and did increase with age.

### 3.2. Effects of Sex and Age on HDL-C and HDL-C Peroxide Content

**Figure 1:**
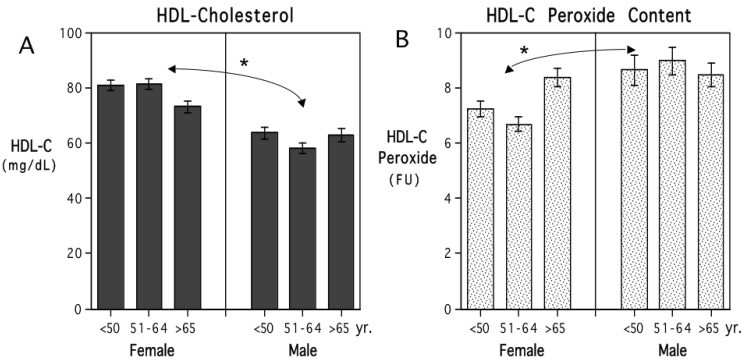
Mean (+/− S.E.) HDL-C and HDL-C peroxide content in female and male adults stratified by age (n = 247 and 216, respectively). Women had significantly higher levels of HDL-C and lower HDL-C peroxide content than men ((**A**,**B**), respectively). Information on other lipids in the panel is provided in Table 2. Participant ages are shown in three categories (<50, 51–64, and >65 years) to illustrate the influence of age, but age was treated as a continuous, parametric variable in the regression models. Asterisk (*) indicates the significant difference between females and males.

As shown in Figure 1, women had significantly higher levels of HDL-C than men, especially among the female participants younger than 50 years of age. There was also a significant effect of sex on HDL-C peroxide content, which was more pronounced in the women younger than 64 years of age. HDL-C levels tended to decline with age whereas the HDL-C peroxide content increased with age.

### 3.3. Predictors of HDL-C Peroxide Content

A hierarchical regression analysis was performed to further explore the influence of age and sex on HDL-C peroxide content, and to probe the additional effects of other factors on this measure of oxidized HDL-C (Table 3). An initial bivariate analysis affirmed the significant effect of sex: being female was associated with lower HDL-C peroxide content (Model 1). When age, which was associated with higher HDL-C peroxide content, was added, the model fit improved (Model 2). The previously detected beneficial effect of being female remained significant. HA1c and CRP, two biomarkers associated with dyslipidemia and HDL-C dysfunction, were then added sequentially to the model. The inclusion of biomarkers indicative of poor glycemic control and inflammatory activity improved the R^2^ value without significantly affecting the *p*-value of the overall model (Models 3 and 4, respectively). They did not lessen the influence of sex on HDL-C peroxide content but reduced the amount of variance accounted for by age. Upon adding waist circumference, which was highly correlated with HDL-C peroxide content, the association between sex and HDL-C peroxide content no longer retained significance (Model 5). However, the overall model’s R^2^ improved. Moreover, this effect was enhanced further by adding non-HDL cholesterol to the model (Model 6). The level of non-HDL cholesterol was directly associated with HDL-C peroxide content, and its inclusion improved the model fit, independent of the effects of gender on HDL-C peroxide content. 

### 3.4. Prevalence of Elevated HDL-C and Its Effect on HDL-Peroxide Content

Many Japanese adults had particularly high levels of HLD-C. Values over 90 mg/dL were categorized as elevated, and the potential influence on HDL-C peroxide content was examined. As shown in Table 4, this categorization yielded two dichotomous subgroups with significantly different mean levels of HDL-C (64.4 vs. 103.6 mg/dL, *p* < 0.0001). In addition, the 77 participants (16.6%) with elevated HDL-C also had significantly lower levels of HDL-C peroxide content (5.8 vs. 8.4 FU, *p* < 0.0001). The individuals with elevated HDL-C did not show overt signs of poor health or hypertension, nor did their circulating levels of CRP and IL-6 differ (Table 4). They also had smaller BMIs and waist circumferences, and lower HA1c levels than did the adults with HDL-C levels below 89 mg/dL.

**Figure 2:**
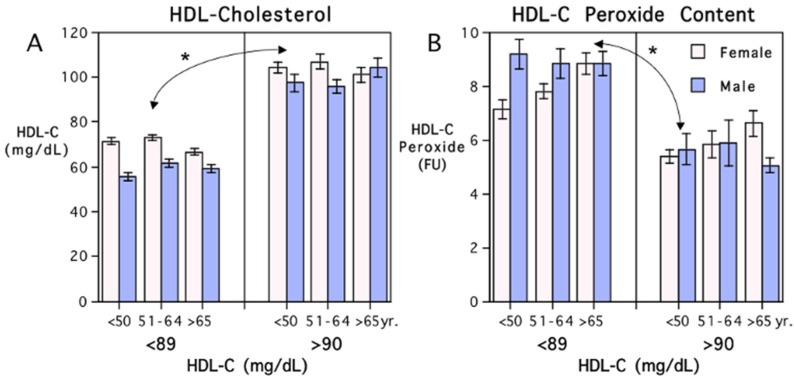
Average levels of HDL-C and HDL-C peroxide content in Japanese adults with HDL-C below 89 mg/dL and with elevated above 90 mg/dL, stratified by age ((**A**,**B**), respectively). Means and S.E. are shown for three age categories. Women had significantly higher levels of HDL-C than men, and a higher percent of the adults with elevated HDL-C were female. Higher HDL-C levels were associated with lower HDL-C peroxide content, and middle-aged women had significantly lower levels of HDL-C peroxide content than men. Asterisk (*) indicates significant difference between participants with HDL-C levels below 89 mg/dL and above 90 mg/dL.

Differences between the participants with elevated HDL-C, compared to adults with HDL-C in more typical ranges, are illustrated in Figure 2. The graphs also show the influence of sex and age on the association between the HDL-C level and HDL-C peroxide content. Although an influence of being female on HDL-C peroxide content is evident, the large effect of elevated HDL-C is predominant. There was some synergism with the effect of sex because participants with elevated HDL-C were more likely to be female (25.1% of the females versus 6.9% of the males had HDL-C over 90 mg/dL). When the participants were subdivided into three age categories from youngest to oldest adults, the pervasive relationship between very high levels of HDL-C and lower HDL-C peroxide content was still evident in both women and men. Delineating the lipid data by both sex and age highlighted that low levels of HDL-C peroxide content were most prominent in younger women. It also revealed that the age-related increases in HDL-C peroxide content were clearer in women than men, a maturational trend that was evident in both the women with lower and with elevated HDL-C level.

### 3.5. Stability of Elevated HDL-C and Lower HDL-C Peroxide Content over Time

**Figure 3:**
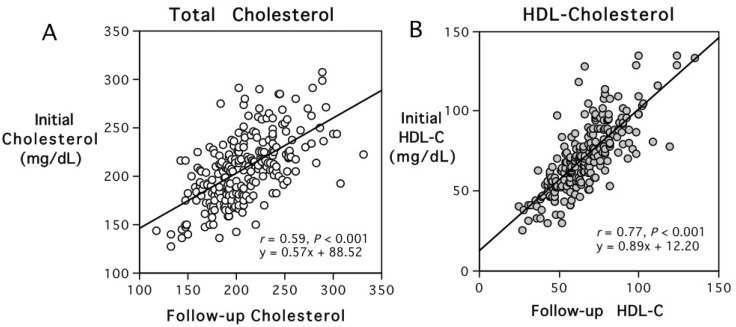
Scatterplots showing the stability of total cholesterol (**A**) and HDL-cholesterol levels (**B**) in the 241 participants evaluated during the initial recruitment phase in 2008 and then at follow-up 5 years later. HDL-C levels are conveyed with gray-fill in the circles.

The persistence of individual differences in HDL-C levels and HDL-C peroxide content was assessed further in the 241 adults who participated in the follow-up analysis. Their mean age at the initial recruitment in 2008 was 55.8 (0.9) years, and they were 60.5 (0.9) years of age at follow-up. Figure 3 shows that their levels of total cholesterol and HDL-C remained similar and were consistent at the two assessments (*r* = 0.59, *p* < 0.001; *r* = 0.77, *p* < 0.001, respectively). Their HDL-C peroxide content values were also significantly correlated at the two assessments (*r* = 0.47, *p* < 0.001). The 43 participants with follow-up samples who had elevated HDL-C (>90 mg/dL) at the first collection also continued to have higher total cholesterol and HDL-C levels and lower HDL-C peroxide content at the second assessment than did the 198 adults with HDL-C values below 89 mg/dL when the first blood was collected (*p* < 0.0001).

## 4. Discussion

Our findings on cholesterol levels in middle-aged and older Tokyo residents align with many previous studies, which showed that the lipid profiles of Japanese adults are generally healthier than those of European populations as well as other East and South Asian countries [42,43,44]. In particular, the prevalence of high levels of HDL-C in Japan is distinctive and contributes to a comparatively high total cholesterol level that is often above 200 mg/dL [45]. Total cholesterol test results in this range are typically considered unhealthy in other populations due to elevated levels of LDL-C and triglycerides. Given the occurrence of so many high total cholesterol and HDL-C values, we verified that the cholesterol values generated by the clinical laboratory in Tokyo were comparable to test results when the sera were tested again at a CLIA-certified clinical laboratory in the United States (Figures S1 and S2). 

In keeping with expectations, women had healthier lipid profiles than men [45,46]. Sex differences in lipid values were associated with the two measures of adiposity, which highlighted the low BMIs and smaller waist circumferences of most Japanese women [47]. In addition, there were sex differences in the levels of the two protein measures of inflammatory activity, CRP and IL-6, as well as in the levels of HA1c, the bioindicator of glycemic control, which can adversely impact lipid metabolism [48,49,50,51,52]. However, it should be reiterated that CRP and IL-6 levels generally tend to be low in both Japanese women and men, and the prevalence of Type 2 diabetes in the MIDJA participants was only 7% [41].

Higher levels of HDL-C were associated with lower HDL-C peroxide content. But it should be acknowledged that there have been mixed findings on whether extremely elevated levels of HDL-C are associated with an increase in ASCVD morbidity and mortality [20,21,22,23,24]. For example, one study of patients with ASCVD found that elevated HDL-C was associated with an increased hazard risk for all-cause mortality, but the association between HDL-C levels and cardiovascular disease was linear, with an increased risk evident only in patients with low HDL-C [24]. Within our study population, comprised primarily of healthy adults, we did not detect the clinical conditions evident in some patients with a hyperalphaliproteinemia diagnosis (HALP) [53]. However, we did not use genotypes to identify the participants with CETP alleles that underlie the elevated HDL-C levels that often occur in many HALP patients. It has been estimated that CETP mutations and the resulting deficiency of CETP may be present in over 30% of Japanese adults who have HDL-C elevated above 80 mg/dL [54,55]. We did not detect any negative consequences of maintaining elevated HDL-C over time among the adults who provided a second blood sample 5 years later. In fact, the adults in the follow-up who had elevated HDL-C above 90 mg/dL had lower blood pressures and HA1c levels. They also had lower BMIs and smaller waist circumferences and tended to have lower CRP and IL-6 levels. 

This follow-up assessment demonstrated the stability of the association between higher levels of HDL-C and lower HDL-C peroxide content. However, it should be noted that in the MIDJA participants, very high HDL-C levels occurred more commonly in women than men. HDL-C levels above 90 mg/dL were found in 25.1% of the women versus 6.9% of the men, a sex difference found by others [56]. There was also a pronounced sex difference in the HDL-C peroxide content, which was most evident in the Japanese women younger than 50 years of age. Given that higher HDL-C levels and low HDL-C peroxide were prominent in the younger women, it may reflect the health-promoting effects of estrogen in premenopausal women [48,49]. The healthier lipid profiles of the younger women contributed to clearer age-related trends in HDL-C and HDL-C peroxide content in the female participants, although the rise in central adiposity among many older Japanese men was associated with their higher HDL-C peroxide content. 

### 4.1. Limitations

While our findings on the predictors of HDL-C level and HDL-C peroxide content are in keeping with the common risk factors for ASCVD, including age, sex, obesity, diabetes, and inflammatory activity [42,43,44,45], we should acknowledge several limitations. First, we did not obtain detailed dietary records. It is known that nutrition, as well as exercise, can have strong effects on lipid metabolism [57,58,59]. In addition, we had reported previously that adherence to more traditional diets and lifestyle practices, including drinking green tea, eating seafood regularly, and partaking in relaxing hot baths, reduced the inflammatory physiology of participants in the MIDJA [60]. The current analysis also did not consider the many psychosocial and economic factors that can influence cholesterol levels and risk for ASCVD [61]. The prevalence of ASCVD and diabetes is also known to vary across cities and to differ between urban and rural areas of Japan, reflecting the important effects of socioeconomic factors. Social processes associated with psychological well-being and stress were a major focus of the MIDJA, so these variables can be integrated into future analyses. We consider the current findings to be formative and foundational to further investigation. 

Importantly, we should also acknowledge another limitation, which is that we did not include paraoxonase-1 (PON-1) in the biomarker panel. Many constituents of HDL are responsible for its role in preventing atherosclerosis, but PON-1 is among the most important of the lipid-modifying features, accounting for its antioxidant activity, anti-inflammatory, anti-thrombotic, and anti-adhesion properties [62]. Several different PON-1 polymorphisms have been identified in the Japanese population and have been linked to a differential risk for atherogenic dyslipidemia, diabetes, diabetic microvascular complications, stroke, and osteoporosis [63,64,65,66,67,68,69]. In addition, PON-1 polymorphisms are associated with a differential sensitivity to salt, which is high in Japanese cuisine, and salt intake contributes to hypertension and risk for stroke [70]. The vulnerability of older Japanese adults to cerebral infarction has also been linked to high triglyceride levels [71,72]. While triglyceride values were determined and were in the typical range for Japanese adults, we did not incorporate them into the current analysis because the levels may have been influenced by not being able to collect blood after an overnight fast.

### 4.2. Conclusions

Notwithstanding these caveats and the need for additional research, to our knowledge, this study is the first to investigate lipid peroxidation of HDL-C among Japanese adults in a large-scale and representative manner. The ability to employ the HDL-C peroxide assay on cryopreserved serum samples made it a useful test for interrogating how HDL-C may respond to and contribute to metabolic disorders, inflammatory conditions, and ASCVD. Because the same assay of HDL-C-peroxide content was also used in a survey of middle-aged and older American adults [34], it provides a unique opportunity to compare HDL-C levels and HDL-C peroxide content in two countries. As shown in Figure S4, Japanese adults typically had higher levels of HDL-C and lower HDL-C peroxide content, suggestive of population-level differences in lipid peroxidation and antioxidant activity. It is also of interest that women in both countries had lower HDL-C peroxide content, a sex difference that was particularly evident in both Japanese and African American women. Furthermore, it was of value to document the cholesterol profiles of adult residents of Tokyo during the period from 2008 to 2013, proximal to when national health surveys were conducted [73]. Several investigators have reported that the continuation of dietary changes in Japan, including the inclusion of more Westernized foods, along with the adiposity that accompanies the sedentary inactivity of modern life, have led to temporal changes in the lipid values seen in Japanese adults across the last several decades [74,75,76]. While our findings need to be confirmed with tests of in vivo function and an assessment of long-term clinical outcomes, and the ex vivo determination of HDL-C peroxide content provides only an indirect measure of HDL-C’s anti-oxidative potential, the findings offer some insight into how HDL-C will respond and function.

### 4.3. Clinical Implications

The findings on HDL-C in Japanese adults also affirm the value of considering race and sex when establishing diagnostic norms and the importance of patient diversity when conducting clinical trials. Elevated levels of HDL-C were found more often among Japanese women, typically without overt clinical symptoms, including hypertension. In addition, while extreme obesity was uncommon, the results conveyed that a negative influence of adiposity on lipid metabolism becomes evident in East Asian adults with a lower BMI and smaller waist circumference than for most European and American adults.

## Figures and Tables

**Table 1:** Number of female and male participants at the first biomarker assessment and the longitudinal follow-up 5 years later. Number of individuals who reported taking prescription medications or supplements that could have cholesterol-lowering effects is also shown.

	Baseline			Follow-Up		
	Female	Male	Total	Female	Male	Total
Participant Number	247	216	463	138	103	241
No/Yes Meds *	30/217	18/198	48/415	16/172	8/95	41/217

* Medicine and supplement use was considered as a covariate in the regression modeling.

**Table 2:** Descriptive summary of demographic and biomarker variables.

			*p* Values	
	Female (n = 247)	Male (216)	Sex	Age **
Age (yr)	55.1 (0.9) *	58.0 (0.9)	0.025	-
BMI (kg/M^2^)	21.7 (0.2)	23.8 (0.2)	0.002	0.0001
WC (cm)	70.9 (0.5)	82.0 (0.6)	0.001	0.0001
HA1c	5.7 (0.03)	5.9 (0.01)	NS	NS
CRP (nmol) ***	5.3 (0.7)	14.1 (2.2)	0.04	NS
IL-6 (pg/mL)	1.5 (0.1)	1.9 (0.2)	0.05	NS
Total Chol (mg/dL)	209.4 (2.3)	202.8 (8.7)	NS	0.0003
HDL-C (mg/dL)	79.0 (1.2)	61.7 (1.2)	0.0001	0.0001
LDL-C (mg/dL)	119.7 (2.3)	118.9 (2.5)	NS	NS
Non-HDL-C (mg/dL)	130.4 (2.3)	143.9 (2.9)	NS	0.004
Triglyceride (mg/dL) ****	109.5 (3.5)	164.0 (7.9)	0.0008	0.05
HDL-C peroxide (FU) *****	7.4 (0.2)	8.7 (0.3)	NS	0.0001
Systolic (mmHg)	120.5 (1.2)	130.3 (1.32)	0.0001	0.0001
Diastolic (mmHg)	74.8 (0.7)	80.3 (0.7)	0.0001	0.0001

* Mean (+S.E.) values. ** Significant effects of Age are shown; the extent of some age effects differed by Sex. *** Mean CRP in metric units was 0.56 (0.07) and 1.48 (0.23) mg/L, respectively). **** TG values may have been influenced by the collection of non-fasted samples. ***** HDL-C peroxide content was quantified in fluorescence units (FU) and calibrated with respect to the level of HDL-C in the sera.

**Table 3:** Hierarchical regression models assessing 6 predictors of HDL-C peroxide content in middle-aged and older Japanese adults.

HDL-C_perox_		Sex (*Male Ref*)	Age	HA1c	CRP	Waist Circum.	Non- HDL-C	Model *p*-Value	Adjusted *R*^2^
Model 1	Coeff	−0.141						8.9 × 10^−5^	0.031
	*P*	8.9 × 10^−5^							
Model 2	Coeff	−0.133	0.003					5.7 × 10^−5^	0.037
	*P*	2.1 × 10^−4^	0.041						
Model 3	Coeff	−0.127	0.002	0.365				4.0 × 10^−5^	0.043
	*P*	4 × 10^−4^	0.201	0.062					
Model 4	Coeff	−0.108	0.001	0.358	0.027			3.0 × 10^−5^	0.047
	*P*	3.6 × 10^−4^	0.330	0.067	0.085				
Model 5	Coeff	0.004	0.007	0.202	0.009	0.011		1.2 × 10^−8^	0.085
	*P*	0.927	0.618	0.301	0.558	1.2 × 10^−5^			
Model 6	Coeff	−0.003	0.000	0.106	0.012	0.009	0.002	8.7 × 10^−11^	0.109
	*P*	0.941	0.852	0.584	0.447	5.3 × 10^−4^	2.9 × 10^−4^		

**Table 4:** Biomarker values in Japanese adults with HDL-C in the typical range (<89 mg/dL) compared to participants who had elevated HDL-C (>90 mg/dL).

	<89 mg/dL	>90 mg/dL	
	**(n = 386)**	**(n = 77)**	***p***
Total Chol (mg/dL)	203.4 (1.9) *	224.7 (3.9)	0.003
HDL-C (mg/dL)	**64.4 (** **0** **.8)**	**103.6 (1.4)**	0.0001
LDL-C (mg/dL)	119.3 (1.7)	115.1 (3.6)	NS
Non-HDL (mg/dL)	138.9 (2.0)	121.1 (4.2)	0.004
Trig (mg/dL)	141.3 (5.0)	102.2 (5.6)	0.05
HDL-C peroxide (FU) **	**8.4 (0.2)**	**5.8 (0.2)**	0.0001
BMI (kg/M^2^)	23.1 (0.2)	20.5 (0.2)	0.0001
WC (cm)	78.0 (2.5)	69.2 (0.8)	0.0001
HA1c (%)	5.9 (0.04)	5.7 (0.04)	0.0019
CRP (nmol)	9.9 (1.3)	7.1 (2.2)	NS
IL-6 (pg/mL)	1.8 (0.1)	1.4 (0.1)	NS
Systolic BP (mmHg)	125.9 (1.0)	120.5 (2.3)	NS
Diastolic BP (mmHg)	78.1 (0.6)	73.8 (1.3)	NS

* Mean +S.E. values are shown. HDL-C and HDL-C peroxide content highlighted in Bold font. ** HDL-C peroxide content was quantified in fluorescence units (FU) and calibrated with respect to the level of HDL-C in the serum sample. HDL-C peroxide content was significantly lower in adults with very high levels of HDL-C.

## Data Availability

Demographic and biological data from the MIDJA project are publicly available at the Inter-university Consortium for Political and Social Research (ICPSR) as part of the MIDUS collection. This public portal provides researchers access to searchable variable-level metadata and longitudinal harmonization information, which can be downloaded as customized datasets and codebooks. For more information about data availability and the MIDJA and MIDUS projects see: http://midus.wisc.edu/data/index.php, accessed on 17 November 2024.

## Supplementary Materials

The following supporting information can be downloaded at: https://www.mdpi.com/2076-3921/13/12/1434/s1, Figure S1: Total Cholesterol Comparison; Figure S2: HDL-C Assay Validation; Figure S3: Glycosylated Hemoglobin (HA1c%) validation; Figure S4: Comparison of HDL-C and HDL-C peroxide content in Japanese and American adults (MIDJA and MIDUS, respectively).
