# Supplementary material for: Healthier Lipid Profiles of Japanese Adults, Especially in Women with Elevated High-Density Lipoprotein Cholesterol (HDL-C), Are Associated with Low HDL-C Peroxide Content
Source: Antioxidants (Basel). 2024 Nov 22;13(12):1434. doi: 10.3390/antiox13121434 (PMC11870043; doi:10.3390/antiox13121434)
Supplement: Supplementary file 1 [file antioxidants-13-01434-s001.zip › antioxidants-3318342-supplementary.pdf]

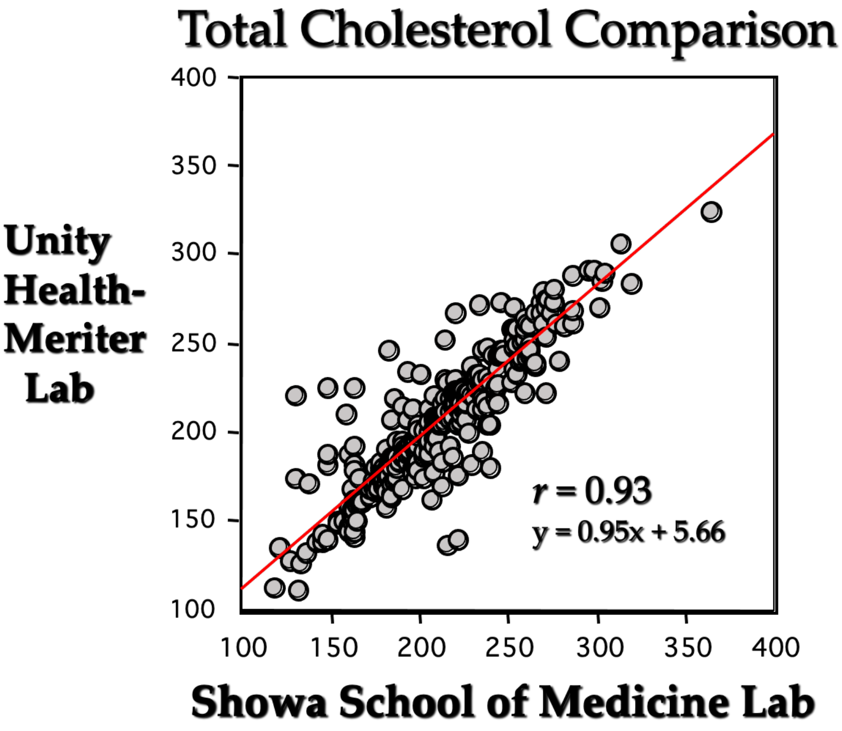

Figure S1. Comparison of Total Cholesterol values in 100 sera from MIDJA study participants, which were tested at both the Showa University School of Medicine laboratory (Tokyo, Japan) and the CLIA-certified Unity-Health Meriter Laboratory (Madison, WI). The results verified the similarity of the lipid test values generated by the two laboratories. The two sets of Total Cholesterol values were highly correlated (r = 0.93, $P < 0.01$), validating the reliability of the test results, including when Total Cholesterol levels were high and quantified above 200 mg/dL.

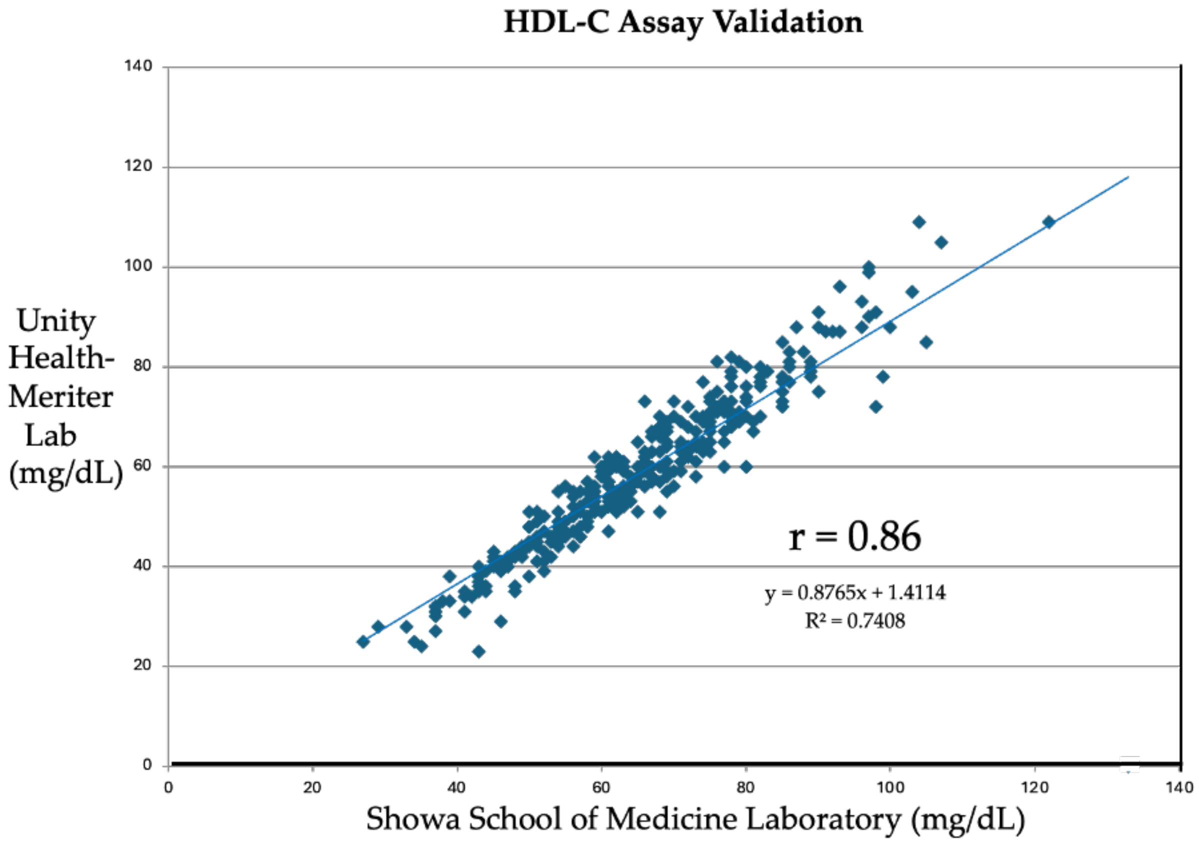

Figure S2. Comparison of HDL-C values in 100 sera from MIDJA study participants, which were tested at both the Showa University School of Medicine laboratory (Tokyo, Japan) and the CLIA-certified Unity-Health Meriter Laboratory (Madison, WI). The lipid test values generated by the two laboratories were highly correlated (r = 0.86, P < 0.01), validating the reliability of the Japanese values and the comparability to diagnostic testing in the United States., The HDL-C levels shown in the figures and tables in the paper are from the clinical laboratory of the Showa University School of Medicine. These values were also used in the formulas when calculating HDL-C peroxide content, which standardized the fluorescence units to 1 mg/dL HDL-C for all specimens (FU/mg/dL HDL-C), the primary unit of quantification for the analyses.

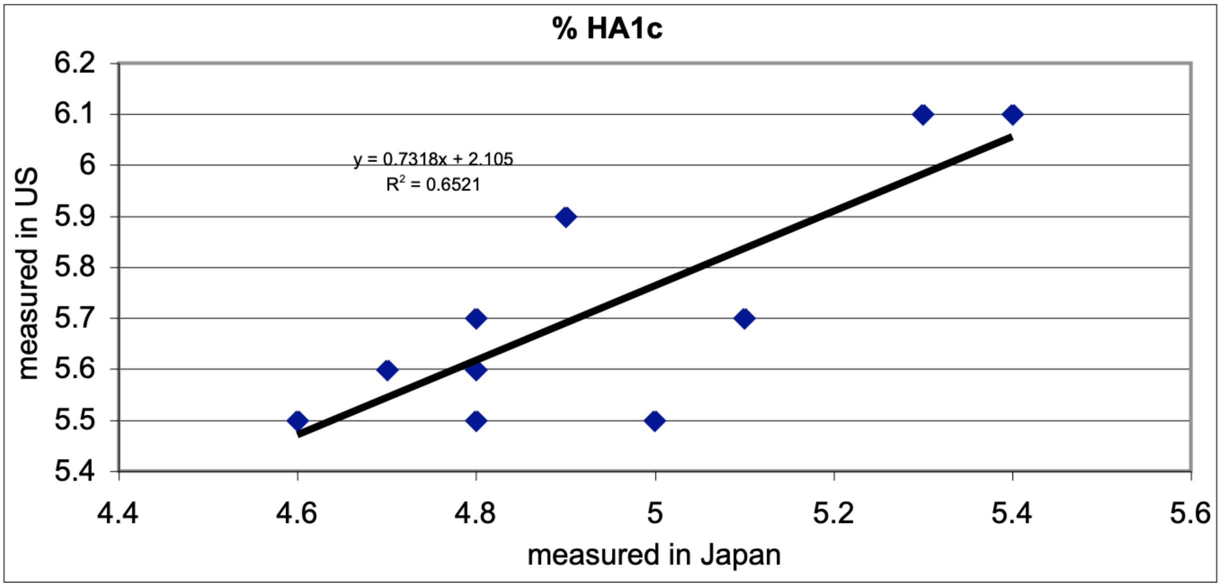

Figure S3. Comparison of glycosylated hemoglobin (HA1c) test results on 10 whole blood samples, which were determined on the collection day at the Showa University School of Medicine laboratory (Tokyo, Japan) and at the Unity Health-Meriter clinical laboratory (Madison, WI) after overnight shipment of aliquots from the same blood on cold refrigerant blocks to the U.S. We and others had established previously that HA1c values remain stable in samples stored at 4 °C for one week. HA1c values at the two labs were highly correlated ($r = 0.81$, $P < 0.05$), although the assay calibration differed. An algorithm was generated to scale HA1c values from the Showa laboratory to match the numerical parameters of American clinical laboratories to be able to compare glycemic control in MIDJA participants to its sister project in the US (Midlife in the US, MIDUS). The relative rankings and position in the HA1c continuum from low-to-high was not affected by the rescaling. After transformation, values above 6.5% were considered indicative of poor glycemic control. Based on HA1c values over 6.5% and participant reporting of physician-diagnosed diabetes, the prevalence of insulin resistance in MIDJA participants was 7.0% [**39**]. Pairwise correlations for the current analysis indicated that higher HA1c values were associated with higher HDL-C peroxide content ($r = 0.135$, $P < 0.01$), conveying that poor glycemic control indicative of insulin resistance accentuated the negative effects of adiposity on lipid peroxidation. The hierarchical regression model examining predictors of HDL-C peroxide concentration included HA1c as a continuous, parametric variable.

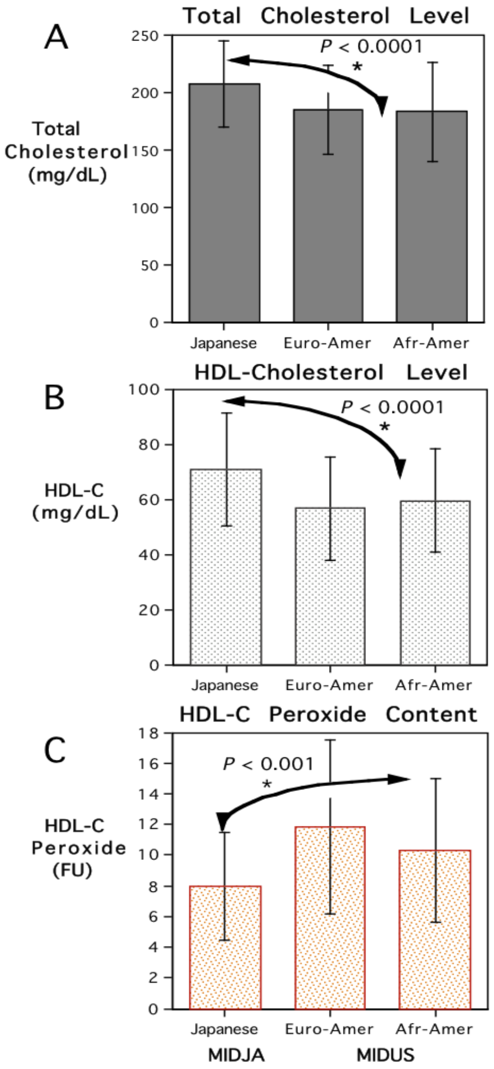

Figure S4. Mean (+/- S.D.) Total Cholesterol (**A**), HDL-C (**B**) and HDL-C peroxide content (**C**) for 463 participants in the MIDJA study compared to mean (+/- S.D.) levels for 1903 white and black American adults in the Midlife in the United States study (MIDUS). Race/ethnic differences were analyzed with one-way ANOVA followed by Scheffe post hoc testing of significant main effects. Total Cholesterol and HDL-C levels were significantly higher ($P < .0001$) and HDL-C peroxide content was lower in Japanese adults (P < 0.001). Total Cholesterol levels for Americans of European family backgrounds and African Americans were representative (mean 182 mg/dL) but reflected the common prescription of lipid-lowering medications in the U.S. when Total Cholesterol exceeds 200 mg/dL. High HDL-C peroxide content in Euro-American adults was driven by higher levels in white men, whereas black women tended to have lower HDL-C peroxide content [**34**]. The large estimates of variance (S.D.) for both Japanese and American adults reflect the many factors that can influence the continuum from healthy to unhealthy lipid profiles.
